# Low-Dose Methylmercury-Induced Apoptosis and Mitochondrial DNA Mutation in Human Embryonic Neural Progenitor Cells

**DOI:** 10.1155/2016/5137042

**Published:** 2016-07-21

**Authors:** Xinjin Wang, Mengling Yan, Lina Zhao, Qing Wu, Chunhua Wu, Xiuli Chang, Zhijun Zhou

**Affiliations:** School of Public Health/Key Laboratory of Public Health Safety of Ministry of Education/WHO Collaborating Center for Occupational Health/Collaborative Innovation Center of Social Risks Governance in Health, Fudan University, Shanghai 200032, China

## Abstract

Methylmercury (MeHg) is a long-lasting organic pollutant primarily found in the aquatic environment. The developing brain is particularly sensitive to MeHg due to reduced proliferation of neural stem cell. Although several mechanisms of MeHg-induced apoptosis have been defined in culture models, it remains unclear whether mitochondrial DNA (mtDNA) mutation is involved in the toxic effect of MeHg, especially in the neural progenitor cells. In the present study, the ReNcell CX cell, a human neural progenitor cells (hNPCs) line, was exposed to nanomolar concentrations of MeHg (≤50 nM). We found that MeHg altered mitochondrial metabolic function and induced apoptosis. In addition, we observed that MeHg induced ROS production in a dose-dependent manner in hNPCs cells, which was associated with significantly increased expressions of ND1, Cytb, and ATP6. To elucidate the mechanism underlying MeHg toxicity on mitochondrial function, we examined the ATP content and mitochondrial membrane potential in MeHg-treated hNPCs. Our study showed that MeHg exposure led to decreased ATP content and reduced mitochondrial membrane potential, which failed to match the expansion in mtDNA copy number, suggesting impaired mtDNA. Collectively, these results demonstrated that MeHg induced toxicity in hNPCs through altering mitochondrial function and inducing oxidative damage to mtDNA.

## 1. Introduction

Methylmercury (MeHg) is a long-lasting organic pollutant primarily found in the aquatic environment. Due to the biomethylation of inorganic mercury released from anthropogenic sources in waterways, MeHg-contaminated seafood is a major source of human exposure [1]. Therefore, residents that depend heavily on fish for food can be exposed to toxic levels of MeHg [2]. Although MeHg is distributed among various tissues after absorption, brain is its main target, especially the developing central nervous system [3]. Some epidemiological studies have shown that motor and cognitive impairments are the most common neurological changes observed in these populations [4, 5]. It is noteworthy that exposure to MeHg during early life period can be associated with subtle brain damage at levels much lower than those affecting the mature brain [6, 7]. MeHg can elicit pivotal events including calcium homeostasis, mitochondrial dysfunction, formation of reactive oxygen species, and induction of cell death by apoptosis or necrosis through MeHg-induced oxidative stress [3, 8]. However, our understanding of the primary critical targets of MeHg, namely, those which trigger MeHg neurotoxicity, remains incipient.

Although the underlying mechanisms of MeHg-induced neurotoxicity are not completely understood, several lines of evidence demonstrate that reactive oxygen species (ROS) generation in mitochondrial respiration chain (MRC) stress represents a critical event related to the neurotoxic effects caused by this toxicant. Some studies have consistently shown that MeHg exposure can cause oxidative damage in macromolecules, such as lipids and DNA [9]. Mitochondrial DNA (mtDNA) is considered more prone to oxidative damage and consequently gets mutations at a higher rate than does nuclear DNA (nDNA) [10, 11]. This is because mtDNA are easily exposed to high levels of ROS generated during respiration [11]. Moreover, mtDNA lacks protective histones with limited capacity for repair of DNA damage [12]. In addition, mtDNA mutagenesis may be closely related to the stability of the organelle and to mitochondria related apoptosis signaling [11]. Indeed, it has been reported that more ROS are generated from mitochondria in ETC-inhibited and mtDNA-damaged cells [12].

In general, mitochondria produce ATP, act as biosensors for oxidative stress, and eventually become effector organelles for programmed cell death. MeHg can induce apoptosis via the classical mitochondrial pathway in neural stem cells which are in oxidative stress [13, 14]. It is commonly believed that MeHg can also damage the mitochondrial membrane potential, interfere with the function of ETC, and influence the production of ATP by reacting with sulfhydryl groups, which are the components of the mitochondrial membrane protein. Alternations in mitochondrial DNA and mitochondrial dysfunction are now emerging as important factors in the etiology of neuropsychiatric disorders [15, 16]. However, few studies have focused on low-dose MeHg-induced alternations in mitochondrial mtDNA and the investigation of the underlying mechanisms involved in low-dose MeHg-induced toxicity is required.

In the present study, we evaluated the effect of low-dose MeHg on human neural progenitor cells (hNPCs). We analyzed the effect of MeHg on cell viability and cell apoptosis by treating the ReNcell CX cell, an hNPCs line, with low concentrations of MeHg (≤50 nM). Because the toxic effects of MeHg are mainly due to a sustained redox-cycling effect, which result in oxidative stress-related insults, we focused primarily on the examination of ROS production and mitochondria function. Moreover, we investigated mitochondrial gene expression and mtDNA damage in MeHg-treated hNPCs. Our findings suggest that low-dose MeHg induced toxicity in hNPCs through altering mitochondrial function and inducing oxidative damage to mtDNA.

## 2. Material and Methods

### 2.1. Cell Culture

Immortalized human neural progenitor cells (ReNcell CX cells) were obtained commercially from Millipore (Temecula, CA). Cells (frozen at passage two) were recovered and cultured on a 100 mm diameter dish (Corning, Inc., Corning, NY) precoated with laminin, using ReNcell NSC Maintenance Medium containing fresh EGF (20 ng/mL; Millipore) and FGF-2 (20 ng/mL; Millipore), as described previously [17].

### 2.2. MeHg Treatment

MeHg was dissolved in DMSO. The hNPCs were seeded at a density of 2.5 × 10^4^/well in laminin-coated 96-well plates. After 24 h incubation, the medium was changed and MeHg (0 nM, 10 nM, and 50 nM) was added followed by another 24 h culture. All experiments were performed in triplicates and repeated at least three times.

### 2.3. Assessment of Mitochondrial Metabolic Function

Mitochondrial metabolic function of hNPCs was determined with the dye methylthiazoletetrazolium (MTT) to formazan [18]. After being treated with MeHg for 20 h, 10 *μ*L MTT solutions were added to each well, followed by incubation for 4 h at 37°C. The optical density (OD) was detected using a microplate reader (Biotek, Synergy HT, USA) at a test wavelength of 490 nm.

### 2.4. Analysis of Apoptosis by Flow Cytometry

MeHg-induced apoptosis was detected using Alexa Fluor® 488 Annexin V/Dead Cell Apoptosis Kit (Life technology), according to the manufacturer's protocol. Briefly, 1 × 10^6^ dissociated cells were washed twice in cold PBS and resuspended in 1x binding buffer. Then the cells were stained with 5 *µ*L Annexin V-FITC and 1 *µ*L PI for 15 min in the dark, followed by flow cytometric analysis (Epics Altra, Beckman Coulter, USA).

### 2.5. Measurement of Reactive Oxygen Species (ROS)

Intracellular ROS production was measured using DCF-DA (Molecular Probes, Beyotime, Jiangsu, China) [17]. The hNPCs were grown to 80% confluence in 3 cm laminin-coated culture dish and treated with MeHg (10 nM and 50 nM) for 24 h. The positive control groups were treated with Rosup for 30 min at 37°C in the dark place. Then the hNPCs were incubated with 100 *µ*L of 1x DCFH-DA/media solution and placed at 37°C for 30 min. Images were captured using the Olympus microscope system. Fluorescence intensity values were reported as the percentage increase of intracellular ROS with respect to control.

### 2.6. Quantitative Real Time PCR

Total cellular RNA was isolated using Trizol reagent (Invitrogen) according to the manufacturer's recommendations, followed by a reverse transcription with cDNA synthesis kit (Thermo). cDNA was synthesized from 2 *µ*g of total RNA using 1 *µ*L of reverse transcriptase and 50 ng/mL oligo (dT). Each qPCR was carried out in triplicate using SYBR Green PCR Master Mix (Applied Biosystems, CA, USA) at one cycle of 95°C for 10 min, 40 cycles of 95°C for 15 s, and 60°C for 1 min on ABI Stepone Plus Real Time PCR Detection System (Applied Biosystems). PCR mix included 1 *µ*L template cDNA, 5 *µ*L SYBR Green PCR Master Mix (Applied Biosystems), 3 *µ*L ddH_2_O, 1 *µ*L (200 nM) of forward and reverse primers mix. Fold changes in the expression of each gene were calculated by a comparative threshold cycle (Ct) method using the formula 2^−(ΔΔCt)^. PCR primer sequences are available in Table 1.

### 2.7. Detection of Mitochondrial Membrane Potential

Mitochondrial membrane potential was measured using JC-1 Mitochondrial Membrane Potential Detection Kit (Beyotime) according to the manufacturer's protocol. Mitochondrial membrane potential was measured using a fluorescence microplate reader (Biotek, Synergy HT, USA), and images were captured using the Olympus microscope system. J-aggregates are detected as red fluorescence and J-monomers are detected as green fluorescence. The mitochondrion-directed fluorescence-sensitive probe JC-1 was used to determine variations in mitochondrial membrane potential (Δ*ψ*m). Briefly, hNPCs (1 × 10^5^ per well of a 96-well plate) were preloaded with JC-1 (2 mg/mL) and stimulated with 10 *μ*M CCCP as a positive control at 37°C for 20 min. Each experiment was determined in triplicate, and results are expressed as means ± SEM.

### 2.8. Determination of ATP Content

The intracellular ATP concentration was determined with an ATP determination kit (Life Technologies) following the manufacturer's instruction. A 10 *µ*L sample or 10 *µ*L ATP standard solutions were added to 90 *µ*L of reaction buffer in a 96-well plate. Each reaction contained 1.25 *µ*g/mL of firefly luciferase, 50 *µ*M D-luciferin, and 1 mM DTT in 1x reaction buffer. After 15-minute incubation, luminescence was measured using a microplate reader (Biotek, Synergy HT, USA). All experiments were run in triplicate, and the background luminescence was subtracted from the measurement. ATP concentrations in experimental samples were calculated from the ATP standard curve. For protein quantification, a colorimetric method (Pierce bicinchoninic acid [BCA] protein assay kit) was carried out according to the manufacturer's instructions.

### 2.9. Analysis of mtDNA Mutations

Total DNA was extracted from cell samples. Mutations in mitochondrial DNA were determined by PCR, cloning, and sequencing, using primers that specifically amplified the NADH dehydrogenase subunit 1 gene (nucleotide position 3307–4262), ATP synthase 6 gene (nucleotide position 8527–9207), cytochrome b gene (nucleotide position 14747–15887), and noncoding control region (nucleotide position 16024–576) of ReNcell CX mtDNA.

### 2.10. Statistical Analysis

Statistical analysis was performed using Stata 10.0 statistic program. Data are shown as mean ± SEM. Multiple group comparisons were carried out by one-way analysis of variance (one-way ANOVA), followed by Bonferroni's post hoc test. A *P* < 0.05 was considered statistically significant.

## 3. Results

### 3.1. MeHg Decreased Mitochondrial Function and Induced Apoptosis in hNPCs

**Figure 1:**
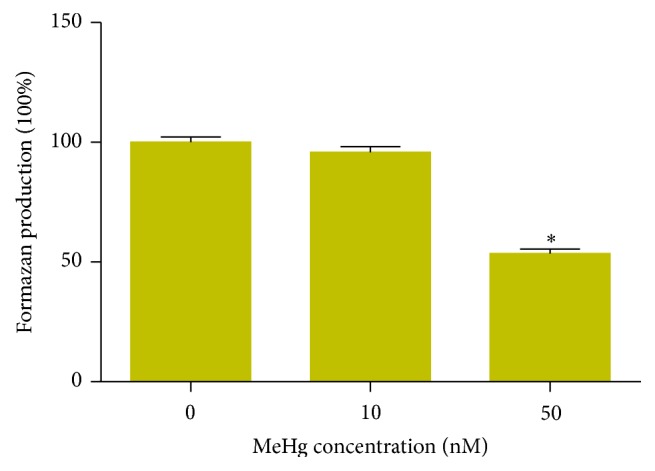
MeHg decreases mitochondrial metabolic function in hNPCs. The results are given as percentage of controls (set to 100%). All experiments were repeated three times (technical triplicates) with biological triplicates (*n* = 3). Bar graphs show mean ± SEM (^*∗*^ *P* < 0.05).

The conversion of methylthiazoletetrazolium to formazan can indicate mitochondrial function and cell viability. The hNPCs were exposed to MeHg at concentrations of 0 nM, 10 nM, and 50 nM for 24 hours. We found that MeHg decreased mitochondrial metabolic function and cell viability in hNPCs in a concentration-dependent manner (Figure 1). At 50 nM MeHg, tetrazolium salt conversion to formazan was reduced to 46.5% of the control value.

**Figure 2:**
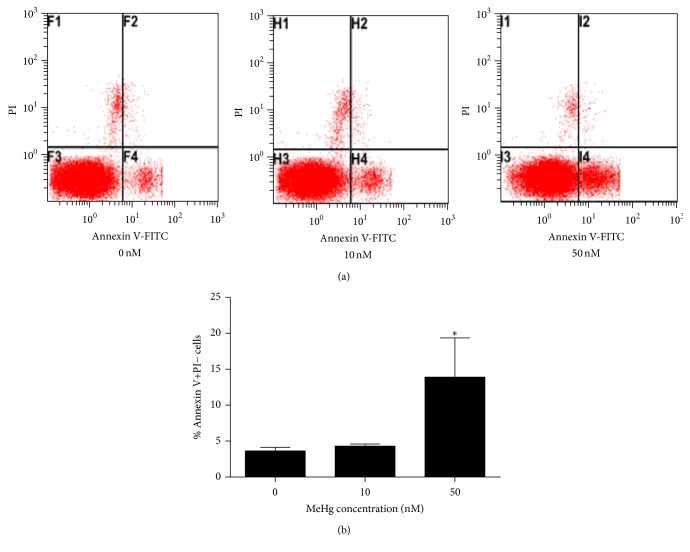
MeHg-induced apoptosis in hNPCs. (a) Cells were stained with Annexin V and PI. Dot plots showed representative staining by flow cytometry, showing the existence of 4 cellular populations: AnnV−/PI−, AnnV+/PI−, AnnV−/PI+, and AnnV+/PI+ (% of each population for all three conditions is provided). (b) The degree ofapoptosis was shown as (or calculated by) the percentage of Annexin V-positive cells in cells treated with MeHg. All experiments were repeated three times (technical triplicates) with biological triplicates (*n* = 3). Bar graphs show mean ± SEM (^*∗*^ *P* < 0.05).

To examine whether MeHg-induced cytotoxicity involves apoptosis, we performed flow cytometric analysis of MeHg-treated ReNcell CX cells by Annexin V-FITC binding assay and PI staining. As shown in Figure 2, Annexin V-PI staining assays detected significantly increased Annexin V-positive apoptotic cells in 50 nM MeHg-treated hNPCs than the control group (0 nM: 3.64 ± 0.49; 10 nM: 4.31 ± 0.29; 50 nM: 13.90 ± 5.47) (Figures 2(a) and 2(b)).

### 3.2. Effect of MeHg on ROS Generation in hNPCs

**Figure 3:**
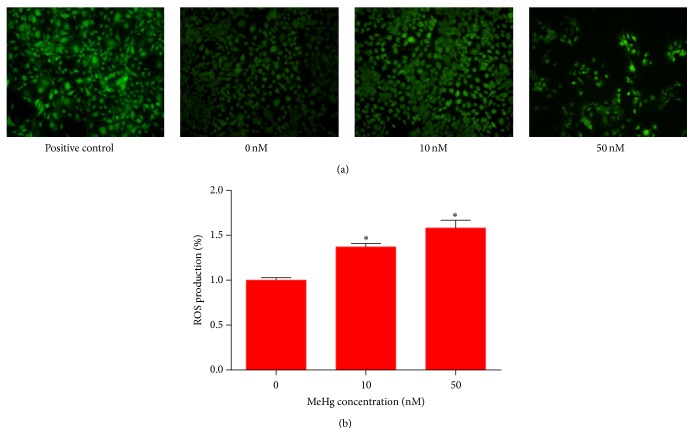
Effect of MeHg on the production of ROS in hNPCs. (a) Cells were treated with 0 nM, 10 nM, and 50 nM MeHg, and the level of ROS was measured by fluorescence microscopic images of DCF-DA signal. (b) The relative ROS levels were presented as fold differences based on control. Results are expressed as means ± SEM (*n* = 3). ^*∗*^ *P* < 0.05 when compared with the corresponding control group (dose: 0 nM).

Accumulating evidence has revealed that ROS are important for the induction of apoptosis. Oxidative stress in particular is considered to be one of the main mechanisms related to MeHg-induced neurotoxicity both* in vivo* and* in vitro* [19]. In this study, the effect of MeHg on the generation of ROS in hNPCs was measured by the DCFH-DA assay (Figure 3(a)). Fluorescence microscope images showed that fluorescence signal of DCF-DA (green) was increased significantly after MeHg treatments (10 nM, 1.47 ± 0.05; 50 nM, 1.69 ± 0.06) (Figure 3(b)).

### 3.3. MeHg Alters Mitochondrial mRNA Transcripts and Mitochondrial Functions in hNPCs

**Figure 4:**
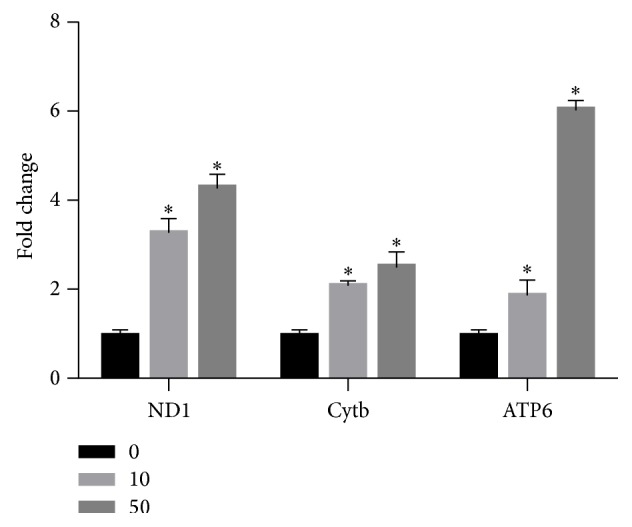
qPCR analysis of ND1, Cytb, and ATP6 mRNA expression levels performed on RNA extracted from hNPCs treated with 0 nM, 10 nM, and 50 nM MeHg. The relative mRNA levels were presented as fold differences based on control. Results are expressed as means ± SEM (*n* = 3). ^*∗*^ *P* < 0.05 when compared with the corresponding control group (dose: 0 nM).

To investigate whether MeHg can change mitochondrial proliferation, we examined the mRNA expression of several genes involved in mtDNA biogenesis and found that MeHg significantly increased the expression of ND1 (fold change: 10 nM, 3.28 ± 0.30; 50 nM, 4.31 ± 0.26), Cytb (fold change: 10 nM, 2.08 ± 0.09; 50 nM, 2.53 ± 0.31), and ATP6 (fold change: 10 nM, 1.88 ± 0.33; 50 nM, 6.03 ± 0.18) (Figure 4).

**Figure 5:**
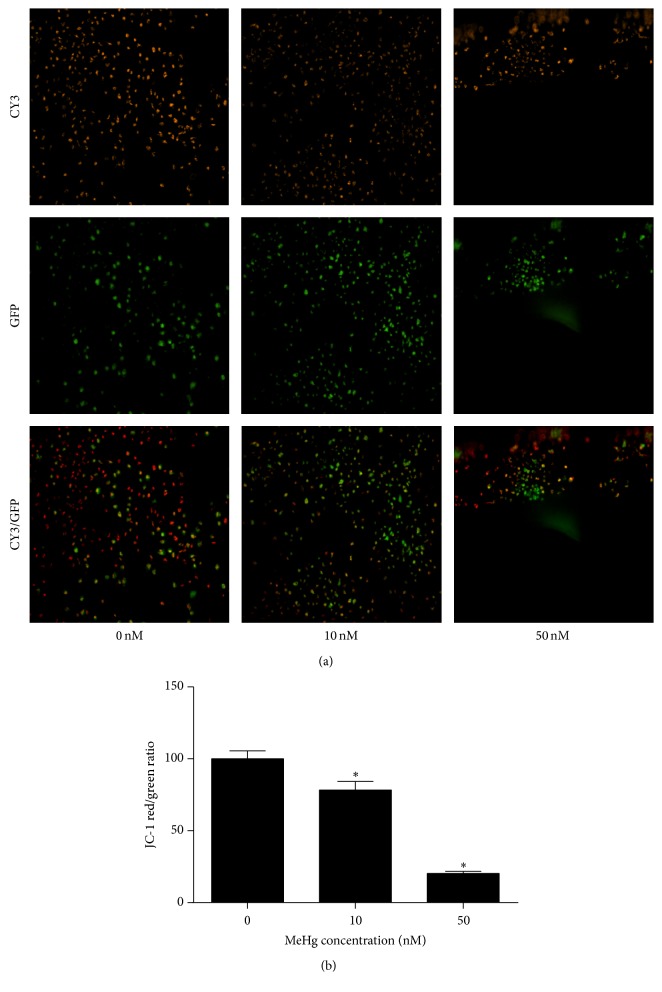
The effect of MeHg on mitochondrial membrane potential in hNPCs. (a) Fluorescence microscopic image of JC-1 signal after MeHg treatment. Images represent typical results of one out of three independent experiments. (b) Mitochondrial membrane potential (Δ*ψ*m) was calculated by the ratio of JC-1 incorporation into mitochondria. Results are expressed as means ± SEM (*n* = 3). ^*∗*^ *P* < 0.05 when compared with the corresponding control group (dose: 0 nM).

**Figure 6:**
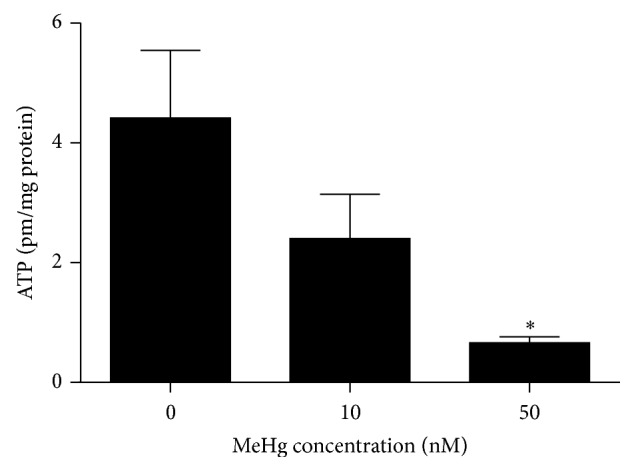
ATP levels were measured in freshly prepared cell extracts treated with various concentrations of MeHg using the ATP determination kit. Results are expressed as means ± SEM (*n* = 3). ^*∗*^ *P* < 0.05 when compared with the corresponding control group (dose: 0 nM).

Moreover, to examine the underlying changes of mitochondrial function induced by MeHg, we then assessed mitochondrial membrane potential (Δ*ψ*m) and ATP cellular content as indicators of mitochondrial function in hNPCs. As compared with untreated cells, MeHg-treated cells revealed a reduced JC-1 aggregation (shift from red/orange fluorescence to green fluorescence), an observation that reflected a drop in Δ*ψ*m (Figures 5(a) and 5(b)). Surprisingly, uncoupled with the increase in expressions of respiratory genes, Figure 6 revealed a dose-dependent loss in ATP content and a significant reduction was observed in 50 nM MeHg-treated cells (0 nM, 4.42 ± 1.13; 10 nM, 2.40 ± 0.74; 50 nM, 0.66 ± 0.10).

### 3.4. MeHg Induced the mtDNA Mutation

We observed that MeHg increased the number of specific point mutations of D-Loop of mtDNA (0 nM, 9; 10 nM, 18; 50 nM, 23), ND1 gene (0 nM, 6; 10 nM, 6; 50 nM, 16), Cytb gene (0 nM, 2; 10 nM, 4; 50 nM, 5), and ATP6 gene (0 nM, 0; 10 nM, 2; 50 nM, 2). The prevalence of the mtDNA mutations found previously unreported in http://www.mitomap.org/ was summarized in Tables 2 [Table tab3] [Table tab4]–5. The data were confirmed by an independent PCR and sequencing analysis, which demonstrated the difference between each group.

## 4. Discussion

In this study, we demonstrated that exposure to low-dose MeHg significantly increased ROS production, reduced mitochondrial metabolic function, inhibited cell viability, and induced apoptosis in hNPCs. In addition, we found a significant loss in Δ*ψ*m and a dramatic decrease in ATP levels after MeHg treatments. Interestingly, MeHg-induced mitochondrial dysfunction could not be rescued by elevated mitochondrial biogenesis, since we observed significant increase of ND1, Cytb, and ATP6 mRNA expression in response to low-dose MeHg exposure. Finally, we first detected that the number of mtDNA (ND1, Cytb, ATP6, and D-Loop) mutations was significantly higher in the 50 nM MeHg-treated group in hNPCs.

The mitochondrion has been recognized as a critical target of MeHg toxicity and a major source of constitutive cellular ROS. Existing evidence supports the role of mitochondrial-mediated oxidative stress to MeHg neurotoxicity, but the majority of investigations have been performed in mature neural cells [20, 21], and the potential role of MeHg-induced ROS in hNPCs is poorly understood. Consistently, we observed a significant increase in ROS generation following MeHg exposure in hNPCs (Figure 3). The ROS could be produced after exposure to lower concentration of MeHg (10 nM) in hNPCs than in neurons (1.5 *μ*M) [22]. Therefore, the high ROS levels observed after MeHg exposure in the present study further support the hypothesis that exposure to low-dose MeHg leads to toxicity in hNPCs. Furthermore, these findings are in agreement with results of several previous* in vivo* and* in vitro* studies from whole brain [23–25]. Thus, the agreement between previous publications and our present findings, gathered in a diversity of preparations, further confirms the use of hNPCs in examining the toxicity following exposure to MeHg.

Mitochondria are maternally inherited and have a primary function of generating ATP via oxidative phosphorylation. Additionally, mitochondria are vital role in the regulation of complex survival signals that determine whether cells live or die and are closely involved in several other functions including cellular differentiation, growth, and cell cycle control [26]. It has been shown that MeHg-induced ROS production occurs with reduced mitochondrial function [27]. In the present study, we observed that MeHg exposure impaired mitochondrial function in hNPCs, demonstrated by a significant loss in Δ*ψ*m at 10 nM MeHg-treated group. Furthermore, we found that ATP levels were dramatically decreased after MeHg exposure, which are consistent with previous studies [27]. Moreover, mitochondria dysfunction was further confirmed by the observation that MeHg treatment significantly reduced the conversion of methylthiazoletetrazolium to formazan (Figure 1). This reaction is mainly catalyzed by succinate dehydrogenase in the mitochondria, although other dehydrogenases can also contribute. Collectively, the findings presented here suggest that MeHg exposure, at the concentration as low as 10 nM, impairs mitochondrial function and lowers cell viability. Meanwhile, we found that 24 h exposure to low doses of MeHg, varied from 10 nM to 50 nM, induced apoptosis of cultured hNPCs. Importantly, these doses of MeHg mimic the environmental exposure and are even below the doses in human cord blood [28]. Since the available evidence from* in vivo* and* in vitro* studies shows that MeHg-induced apoptosis is mediated through the mitochondrial-dependent pathway in neural developmental toxicity [29], the present findings suggest that mitochondria are the primary target for MeHg and that ROS production could be a consequence of damage to the mitochondria rather than being an initiator of mitochondrial damage.

It has been widely reported that alterations in intracellular ROS level are often associated with changes in mitochondrial abundance and the expression of respiratory genes [10]. Indeed, our results showed that low-dose MeHg increased mtDNA transcription in hNPCs, with increased mRNA expression of ND1, Cytb, and ATP6. Interestingly, we observed a decrease in ATP levels after MeHg exposure, indicating that the increased mitochondria biogenesis fails to compensate for the decline of mitochondrial respiratory function. The relationship between oxidative stress and mitochondria function is complicated. Oxidative stress-induced increase in mitochondrial abundance and expressions of respiratory genes may compensate for the decline of mitochondrial respiratory function. Simultaneously, ROS would also be generated from the increased mitochondria and thus cause much more oxidative damage to mitochondrial macromolecules, such as lipids and DNA, and consequently lead the cell to enter the process of senescence or apoptosis [30, 31].

Mitochondrial DNA is the critical importance for preserving mitochondrial respiratory function. Human mtDNA conserve a small genome, which is 16,569 bp in length and encodes 13 polypeptides of ETC, 22 tRNAs, and 2 rRNAs located in the inner mitochondrial membrane (IMM) matrix [32]. It is noteworthy that mtDNA is highly susceptible to oxidative damage compared to nDNA, which is due to its close proximity to the cell's main source of ROS production, lack of a histone protection, and relatively less efficient DNA repair mechanisms [11]. Indeed, oxidative damage in mtDNA is a mutation rate that is 10-fold greater as compared to nDNA [33]. Several studies have indicated the associations between genetic damage induced by MeHg and alterations of the cellular redox status, and it is conceivable that ROS induced DNA damage plays a role in the adverse health effects [34, 35]. In the present study, we first observed that the number of mtDNA (ND1, Cytb, ATP6, and D-Loop) mutations was higher in the 50 nM MeHg-treated group compared to the control, especially for mutations in ND1 and D-Loop in hNPCs (Tables 2 [Table tab3] [Table tab4]–5). Meanwhile, we observed that low-dose MeHg increased mtDNA transcription with increased ROS in hNPCs. The increased copy number of mtDNA is suggested as a result of the feedback response that compensates for defective mitochondria bearing impaired respiratory chain or mutated mtDNA by ROS [36]. Conversely, an increase in expressions of respiratory genes could contribute to further oxidative stress leading to mtDNA instability, mitochondrial dysfunction, and oxidative damage [37]. Moderate oxidative stress may lead to the increase of mitochondrial abundance and mtDNA content [10]. Nevertheless, chronic exposure to oxidative stress may cause severe oxidative damage and agitate the stress response of the target cells [38]. Mitochondrial DNA damage may trigger cell deaths, which result from compromised bioenergetic function, genetic and protein instability, and increased ROS generation [39]. As expected, mtDNA instability can elicit mitochondrial dysfunction, including the loss in the mitochondrial membrane potential (Δ*ψ*m) and release of proapoptogenic agents, which drives disease formation, aging, and tumorigenesis [40]. Therefore, low-dose MeHg-induced long-term oxidative stress in mitochondria may be a contributory factor to the somatic mtDNA instability and thereby leads to a decline in mitochondrial respiratory function. Moreover, we have to point out that we do not directly detect ROS in mitochondria. In order to clear link between ROS generation and mitochondrial damage, further studies should be required.

In conclusion, our study provides evidence that low-dose MeHg exposure promotes mitochondrial gene upregulation in hNPCs, which, in turn, contributes to the respiratory chain generating more ROS. Excess ROS further damages mtDNA, leading to ATP depletion and membrane potential (Δ*ψ*m) loss. Ultimately, the additive or cooperative mechanisms of cellular disruptions caused by MeHg lead to cellular dysfunction and cell death.

## Figures and Tables

**Table 1:** PCR primer sequences.

Primer name	Forward	Reverse
ND1	ACCCCCTAGGAATCACCTCC	GCCTAGGAGGTCTGGTGAGA
Cytb	CGATTCCGCTACGACCAACT	AGGTTTGAGGGGGAATGCTG
ATP6	CTGTTCGCTTCATTCATTGC	AGTCATTGTTGGGTGGTGATT
GAPDH	GTCTCCTCTGACTTCAACAGCG	ACCACCCTGTTGCTGTAGCCAA

**Table 2:** Specific point mutations of ND1 gene found after treatment with MeHg in hNPCs.

Group	Gene site	Mutation type
0 nmol/L	3450	del(C)
	3487	C → G
	3653	T → C
	3728	A → G
	4018	A → G
	4219	T → C

10 nmol/L	3319	A → C
	3321	C → A
	3323	T → C
	3415	C → A
	3704	T → C
	3791	T → C

50 nmol/L	3316	del(G)
	3346	C → G
	3376	G → A
	3471	C → T
	3495	C → T
	3497	C → G
	3501	A → C
	3510:3511	ins (AT)
	3547:3548	ins (AT)
	3556	C → T
	3557:3558	ins (T)
	3562:3563	ins (AT)
	3579	del(A)
	4043	G → A
	4103	T → C

**Table 3 tab3:** Specific point mutations of Cytb gene found after treatment with MeHg in hNPCs.

Group	Gene site	Mutation type
0 nmol/L	15611	T → C
	15719	T → A

10 nmol/L	15017	T → C
	15251	T → C
	15682	del(A)
	15740	del(CT)

50 nmol/L	15082	C → G
	15621	T → C
	15679	del(A)
	15702	del(C)
	15743	del(C)

**Table 4 tab4:** Specific point mutations of ATP6 gene found after treatment with MeHg in hNPCs.

Group	Gene site	Mutation type
10 nmol/L	8636	T → G
	9048	T → G

50 nmol/L	9056	C → T
	9166	T → C

**Table 5:** Specific point mutations of D-Loop gene found after treatment with MeHg in hNPCs.

Group	Gene site	Mutation type
0 nmol/L	349:350	ins (C)
	378	del(A)
	382:383	ins (C)
	387	del(C)
	444:445	ins (A)

10 nmol/L	302	A → G
	353:354	del(CC)
	372:373	del(TA)
	378	del(C)
	385	del(A)
	403	ins (T)
	410	G → C
	413	G → T
	493	A → C
	505	del(C)
	16125	G → A

50 nmol/L	123	A → G
	246:247	ins (TG)
	324	del(C)
	384	del(A)
	394:395	del(CA)
	397	A → T
	424:425	del(TA)
	430	T → A
	440	del(A)
	442	T → C
	457	G → C
	469	del(C)
	478	del(A)
	490:491	del(AC)
	493	A → C
